# An exploration into the quality of life of women treated for cervical cancer

**DOI:** 10.4102/curationis.v42i1.1982

**Published:** 2019-05-28

**Authors:** Caroline Sabulei, Johanna E. Maree

**Affiliations:** 1 Department of Nursing Education, University of the Witwatersrand, Johannesburg, South Africa

**Keywords:** cervical cancer, quality of life, radiotherapy, South Africa, cross sectional

## Abstract

**Background:**

Cervical cancer mainly occurs among women from the developing world, and women face unique challenges in terms of their disease and treatment. Most women present with advanced cervical cancer and receive the standard curative treatment with external beam radiotherapy and brachytherapy with or without chemotherapy.

**Objectives:**

To describe the quality of life (QOL) of women treated for cervical cancer during treatment (M0), at 6 months after completing treatment (M6) and at 12 months after treatment (M12).

**Methods:**

A cross-sectional design, calculated sample size (*n* = 153) and convenience sampling were used. Data were collected through structured interviews, and the EORTC QLQ-C30 and EORTC QLQ CX24 served as data collection instruments. Descriptive statistics were used to analyse the data, and the Kruskal–Wallis H test was used to compare the mean responses across the groups (*p* ≤ 0.05).

**Results:**

The mean age of the respondents was 50.6 years (standard deviation [SD] 11.9). The global health status improved significantly in contrast with the functional scores. Financial difficulties were rampant, especially during the treatment phase. Insomnia and urinary frequency were the most cumbersome problems and remained so even after treatment.

**Conclusions:**

Despite an improvement in the global health, cervical cancer and its treatment had a negative influence on the QOL in all domains of lives of these women. Assessing the QOL of patients during treatment and follow-up visits would allow nurses to develop interventions to address distressing problems timeously. In addition, Africa’s nurses should assess social functioning and develop programmes to prevent social dysfunction.

## Introduction

The significance of this study derives from the fact that best practices focus on the prevention and treatment of cervical cancer and little attention has been given to the quality of life (QOL) of women living with this condition. Although it is known that cancer and its treatment influences QOL, studies focusing on this phenomenon in the African population appear to be sparse (Maree, Herbert & Huiskamp 2017). Cervical cancer is primarily a disease of women living in the developing world (World Health Organization 2006), and the challenges they face in terms of access to health care, financial resources and education are factors that could influence the QOL of women treated for this disease. This study adds to the body of knowledge on this phenomenon in its attempt to describe the QOL of these patients.

## Background and literature review

Cervical cancer is the fourth most common cancer in women worldwide. According to the latest Globocan statistics (International Agency for Research on Cancer and World Health Organization 2014), approximately 528 000 women were newly diagnosed with cervical cancer in 2012, while 266 000 women died from this disease. The largest burden of the disease (85%) and most of the deaths (87%) occurred in the developing world. Southern Africa has one of the highest incidences of cervical cancer similar to Eastern and Middle Africa and Melanesia. In South Africa, cervical cancer is the second most common cancer in women after breast cancer but the most common cancer in black women, being responsible for approximately 30% of all cancers diagnosed in this group (National Institute for Occupational Health 2018).

Cervical cancer is primarily caused by persistent infection with one of the oncogenic types of human papilloma virus (HPV), most commonly type 16 and 18, which is found in 70% of all cervical cancers. Cervical cancer develops slowly, usually taking 10–20 years for mild dysplasia to develop into cancer (World Health Organization 2006). However, women infected with HIV, which is epidemic in South Africa, can develop cervical cancer 10 years before their counterparts who are HIV negative (Snyman 2013). Despite this, cervical cancer is preventable by means of screening (World Health Organization 2006). In South Africa, screening is available at primary health clinics free of cost, but screening coverage is as low as 13% (Snyman 2013), resulting in most women presenting with advanced disease (Snyman & Herbst 2013). According to the International Atomic Energy Agency (2012), the stages of advanced cervical cancer are IB2 and IIA2 ≥ 4cm – IIB – IIIA – IIIB – IVA, and they require standard curative treatment with external beam radiation and brachytherapy with or without concomitant chemotherapy, most commonly cisplatin. Cisplatin is administered in five weekly doses at the same time the patient receives external beam radiation.

Living with cervical cancer is not easy. The lives women knew before they are diagnosed change (Chan & Molassiotis 2001), and they are subjected to short-term and long-term physical, psychosocial and sexual problems, which have a negative influence on their QOL (Ancuţa et al. 2012). For instance, receiving brachytherapy is a negative, humiliating experience causing pain and fear (Dzaka & Maree 2016), and women struggle with chronic fatigue; chronic pain; vaginal, bladder and bowel problems; sexual problems; financial hardship; social problems; body image; and role changes after treatment (Chan & Molassiotis 2001; Ntinga & Maree 2015). Pilkington and Mitchell (2004) agree that cancer can dramatically change a person’s life – especially gynaecologic cancer and its treatment as it influences women’s experience of themselves as sexual beings.

Quality of life is a multidimensional concept (Goker et al. 2011; Maree & Van Rensburg 2016) without a common definition or standard of measurement (Ferrans 1990). The multidimensional aspect of QOL, according to Cella (1994), refers to the broad coverage of content, which includes physical, functional, emotional and social well-being. Ferrans (1990) views the multidimensionality of QOL as the ability to lead a normal life, happiness and satisfaction, achievement of personal goals, social utility and natural capacity. Authors (Cella 1994; Maree & Van Rensburg 2016) agree that QOL is subjective and that the QOL judgement can only be made by the person living the life. QOL is also non-static and can change over time (Olschewski et al. 1994), which means that patients in the same situation and stage of illness can judge their QOL differently.

Quality of life has become an important consideration in evaluating the efficiency of health care and particularly significant in the field of cancer care, as treatments are often incapacitating but at the same time increase long-term survival (Pilkington & Mitchell 2004). If patients’ QOL is measured on a regular basis, using an appropriate instrument, the data have the potential to enhance cancer care decisions (Morris, Perez & McNoe 1997) and improve patient outcomes.

### Conceptual framework

The Wilson and Cleary (1995) model for health-related QOL, adapted by Ferrans and others (2005), underpinned this study. This model focuses on the measurement of five types of patient outcomes, biological function, symptoms, functional status, general health perceptions and overall QOL, which are influenced by the characteristics of the individual and the environment. Biological health refers to the processes that support life on cellular, molecular and organ levels. Serious life-threatening diseases, such as cervical cancer, alter the biological function and influence all the aspects of health, including functional status and QOL.

Symptoms refer to the person’s perception of an abnormal physical, psychological and psychophysical state (Ferrans et al. 2005). According to Ferrans and colleagues (2005), complex interactions with individual and environmental factors, such as knowledge, personality and relations with health-care providers, shape the experience, evaluation and interpretation of symptoms. Various measures, such as global instruments, disease-specific and symptom-specific instruments, are used to measure symptoms. The instruments selected for this study were the EORTC QOL-C30, which was developed for all cancer patients (Aaronson et al. 1993), and the EORTC QLQ CX24, which is cervical cancer specific (Greimel et al. 2006); both instruments include symptom scales.

Functional status refers to the ability of a person to perform their normal activities of daily living to provide for their basic needs, fulfil their usual roles and maintain health and well-being (American Thoracic Society 2007). Wilson and Cleary (1995) describe the functional domains as physical, social, role and emotional functioning, which is investigated by means of the questionnaires used in this study. Ferrans and colleagues (2005) add to the functional status by focusing on the function that remains. However, this study wished to present the functions that remained, and those compromised, to find possible solutions.

General health perceptions are subjective and are presentable as a synthesis of all aspects of life or the outcomes of a single question. Gender influences general health perception in terms of conditions considered and negative emotions experienced (Ferrans et al. 2005). This study had no gender influences because of investigating a gender-specific disease. Overall, QOL is the final component of the model. Similar to general health perception, QOL is subjective and reflects how satisfied a person is with their life as a whole. Quality of life is an individual construct influenced by the patient’s values and preferences. QOL consists of various domains; however, authors differ in terms of the number of the domains. The selected instruments investigate various domains, which include functioning, finances and symptoms. In addition, the EORTC QOL-C30 asks two questions, one focusing on overall health and the other focusing on overall QOL during the past week to present global health status/QOL (EORTC 2001).

### Research problem and objectives of the study

The research problem for the study relates to the QOL of women diagnosed with cervical cancer who received standard curative treatment. The objective of the study was to describe the QOL of women treated for cervical cancer at an academic hospital in Gauteng during treatment (M0), at 6 months after completing treatment (M6) and at 12 months after treatment (M12).

## Research methods and design

### Study design

A cross-sectional design was selected for the study as it allows researchers to investigate time-related phenomena by collecting data from different groups at one point in time and comparing the responses (Polit & Beck 2014).

### Setting

The setting was an academic hospital in Gauteng Province, South Africa. This specialist hospital is part of the public health care service, serving approximately 80% of the population who do not have health insurance. Services are free of charge to the patients as the cost is carried by a National Tertiary Service Grant and funds allocated to the province. The hospital hosts 1088 beds and various outpatient units, and serves as specialist referral hospital for regional hospitals and neighbouring provinces. It also serves as clinical learning platform for nurses, medical practitioners and other health care professionals (Gauteng Province 2016). The adult oncology units are recognised as centres of excellence, and approximately 3500 patients are treated at the Department of Radiation Oncology, the largest oncology unit in the country, annually (SAnews.gov.za. 2014, Gauteng Province S.A.). The department has a chemotherapy room to accommodate patients receiving concomitant chemotherapy and a resting room for patients who need bed rest as they wait to be treated. Most of the patients treated with radiotherapy receive treatment as outpatients, but those who are too ill to travel to the hospital are admitted to the ward designated for radiotherapy patients. Approximately 662–721 women are treated for cervical cancer annually and about 360 are reviewed each month after completing treatment (Dzaka & Maree 2016; Msadabwe 2009). Patients treated for cervical cancer are reviewed 6 weeks after completion of treatment, thereafter every 3 months for 2 years, then every 6 months for 2 years and then annually.

### Population and sampling

The study population consisted of all women diagnosed with cervical cancer treated at the study setting. The inclusion criteria were 18 years and older and receiving the standard curative treatment for cervical cancer at the time of data collection, or 6 or 12 months after treatment. The sample size was calculated with the assistance of a statistician. A finite population correction was used where the initial sample size (*no*) was 157 and the target population (*N*) was 7735.

The formula used was:

$$n=\frac{no}{\left\{1+\left[\frac{no-1}{N}\right]\right\}}$$

which resulted in a sample size of 153 (*n* = 153), thus 51 (*n* = 51) in each of the groups. The total sample (*n* = 153) was determined to achieve a confidence level of 95%. Convenience sampling was used to select the sample as it allowed the researchers to select women who were readily available (Polit & Beck 2014).

### Data collection

Structured interviews (Polit & Beck 2014) were used to collect the data as they enabled the research team to include women from all educational levels. A demographic data sheet and two instruments, the EORTC QLQ-C30 (version 3) and EORTC QLQ CX24, served as data collection instruments with permission. The patient’s file provided the information on the stage of disease. The EORTC QLQ-C30 investigates the quality of life in all cancer patients, while EORTC QLQ CX24 is specific to cervical cancer. The EORTC QLQ-C30 asks 30 questions; 28 questions focus on the functional and physical domains and use a 4-point Likert scale, while the last two questions use a 7-point Likert scale and focus on overall health. The EORTC QLQ CX24 asks 24 questions on functional, symptomatic and global health status. The functional domain is further divided into five parts focusing on physical, role, emotional, cognitive and social functioning. A 4-point Likert scale is used to indicate the answers.

The data were collected from June to August 2015, after ethical clearance was obtained from the University. Women waiting for their scheduled medical review or treatment were recruited either while waiting for their appointments or afterwards when waiting for transport. Participation was voluntary and informed consent, in writing, was obtained before the data were collected. An information leaflet was handed to the women and all who were recruited were willing to participate in the study.

### Data management and analyses

The completed questionnaires were placed in a sealed envelope and numbered sequentially. The data were captured on an Excel spreadsheet and analysed by means of the SPSS 22.0 computer program, with the assistance of a statistician. The scoring procedures of the instruments guided the analysis. A high score for global health and the functional scales represents a high level of functioning, but a high score for the symptom scales and financial burden represents a high problem level.

Descriptive statistics (Polit & Beck 2014) were used to describe and synthesise the data, and the Kruskal–Wallis H test was used to compare the mean responses of the different study groups, level of significance *p* ≤ 0.05.

### Validity and reliability

Validity refers to the degree to which an instrument measures what it’s supposed to measure, while reliability focuses on the accuracy and consistency of an instrument (Polit & Beck 2014). Measures implemented to enhance the validity and reliability of the results included obtaining the participation and cooperation of the Management of the Hospital and Radiation Oncology Department which ensured access to the patients, using validated instruments (Aaronson et al. 1993; Du Toit & Nel 2012) and statistical support. In addition, the first author collected the data thus enhancing consistency.

### Ethical considerations

Ethical clearance was obtained from the University of the Witwatersrand (M150441).

## Results

The ages of the sample (*n* = 153) ranged from 30 to 79 years, with a mean age of 50.6 years standard deviation (SD) ± 11.9. Nearly half (46.4%; *n* = 71) were single and unemployed (48.4%; *n* = 74), and 50.3% (*n* = 77) suffered from stage IIIA and more advanced disease (Table 1).

**TABLE 1:** Demographic characteristics of the respondents (*n* = 153).

Variables	M0 (*n* = 51)		M6 (*n* = 51)		M12 (*n* = 51)		Total	
	Frequency (*n*)	Percentage (%)	Frequency (*n*)	Percentage (%)	Frequency (*n*)	Percentage (%)	Frequency (*n*)	Percentage (%)
**Age groups (years)**								
30–39	11	21.6	12	23.5	12	23.5	35	22.9
40–49	17	33.3	16	31.4	16	31.4	49	32.0
50–59	8	15.7	8	15.7	11	21.6	27	17.6
60–69	12	23.5	15	29.4	8	15.7	35	22.9
70–79	3	5.9	0	0.0	4	7.8	7	4.6
**Marital status**								
Single	21	41.2	25	49.0	25	49.0	71	46.4
Married	16	31.2	14	27.5	18	35.3	48	31.4
Divorced	5	9.8	4	7.8	4	7.8	13	8.5
Widowed	9	17.6	8	15.7	4	7.8	21	13.7
**Employment status**								
Unemployed	23	45.1	25	49.0	26	51.0	74	48.4
Part-time employed	5	9.8	7	13.7	5	9.8	17	11.1
Full-time employed	14	27.5	11	21.6	11	21.6	36	23.5
Pensioners	9	17.5	8	15.7	9	17.6	26	17.0
**Stage of disease**								
IA	2	3.9	1	2.0	0	0	3	2.0
IB	3	5.9	1	2.0	1	2.0	5	3.2
IIA	12	23.5	6	11.7	11	21.6	29	19.0
IIB	14	27.5	16	31.4	9	17.6	39	25.5
IIIA	8	15.7	15	29.4	17	33.3	40	26.1
IIIB	11	21.5	12	23.5	13	25.5	36	23.5
IVA	1	2.0	0	0	0	0	1	0.7

When calculating the global health status of the three groups, it was found that the M12 group had the better health status. A Kruskal–Wallis H test showed that the global health status was statistically significant among the study groups (Table 2). A *post hoc* analysis for significance indicated there was a statistically significant difference in the global health status (*p* = 0.013) between study groups one (M0) and two (M6), between groups one (M0) and three (M12) (*p* = 0.000), and between groups two and three (*p* = 0.024).

**TABLE 2:** Global health status across the groups (*n* = 153).

Variables	Mean ranks			Overall mean	Standard deviation	*p*
	M0 (*n* = 51)	M6 (*n* = 51)	M12 (*n* = 51)			
Global health status	57.5	69.8	82.4	69.9	26.4	0.000*

* , statistically significant, *p* ≤ 0.05.

Functioning, consisting of role, physical, cognitive emotional and social, was also investigated. Role functioning scored the highest overall mean (*m* = 82.0), while social functioning scored the lowest overall mean (*m* = 60.5). In addition, social functioning had the lowest mean score in M0 compared to all the other functioning scores across the different groups. Kruskal–Wallis H tests showed there was no statistically significant difference between the role, physical, cognitive, emotional and social functioning scores among the three study groups. Financial difficulties had the highest mean score in M0 (*m* = 81.7), and a *post hoc* analysis indicated a statistically significant difference between groups one and two (*p* = 0.000) and between groups one and three (*p* = 0.001) (Table 3).

**TABLE 3:** Functioning and financial difficulties across the groups (*n* = 153).

Variables	Mean ranks			Overall mean	Standard deviation	*p*
	M0 (*n* = 51)	M6 (*n* = 51)	M12 (*n* = 51)			
Role functioning	76.8	84.3	85.0	82.0	22.2	0.146
Physical functioning	77.8	84.3	83.4	81.8	17.7	0.068
Cognitive functioning	81.0	85.3	76.5	80.9	21.2	0.312
Emotional functioning	75.7	81.2	85.1	80.7	22.9	0.289
Social functioning	59.5	61.8	60.1	60.5	32.7	0.829
Financial difficulties	81.7	52.9	60.8	65.1	39.4	0.000*

* , statistically significant, *p* ≤ 0.05.

**TABLE 4:** General symptoms reported per group (*n* = 153).

Variables	Mean ranks			Overall mean	Standard deviation	*p*
	M0 (*n* = 51)	M6 (*n* = 51)	M12 (*n* = 51)			
Fatigue	42.0	27.5	20.5	30.0	26.6	0.000*
Pain	38.2	31.4	24.8	31.5	30.7	0.168
Insomnia	47.7	30.7	22.2	33.6	39.8	0.002*
Dyspnoea	18.3	9.8	3.3	10.5	21.8	0.003*
Swelling of the feet	5.2	9.2	35.3	16.6	26.2	0.000*
Tingling of the hands or feet	25.5	29.4	30.1	28.3	25.9	0.545

* , statistically significant, *p* ≤ 0.05.

When investigating the general problems, consisting of fatigue, pain, insomnia, dyspnoea, swelling of the feet and tingling of the hands of feet, dyspnoea had the lowest overall mean (*m* = 10.5) and insomnia had the highest overall mean (*m* = 33.6). Kruskal–Wallis H tests showed there was a statistically significant difference in fatigue, insomnia, dyspnoea, swelling of the feet and financial difficulties, but no statistically significant differences were found in pain and tingling of the hands or feet. A *post hoc* analysis indicated there was a statistically significant difference in fatigue between groups one and two (*p* = 0.003) and between groups one and three (*p* = 0.000). There was a statistically significant difference in insomnia between groups one and two (*p* = 0.024) and between groups one and three (*p* = 0.002). With regard to dyspnoea, the study revealed a significant difference in groups one and three (*p* = 0.001). Groups one and three had significant differences (*p* = 0.000) in swelling of feet as well as groups two and three (*p* = 0.000).

Gastrointestinal, urologic and gynaecologic symptoms were also investigated (Table 5). With regard to the gastrointestinal symptoms, nausea and vomiting had the lowest overall mean (*m* = 24.8), while diarrhoea had the highest mean score (*m* = 22.7). A statistically significant difference was found in diarrhoea, appetite loss, nausea and vomiting. A *post hoc* analysis showed a statistically significant difference between groups one and three (*p* = 0.000) and between groups two and three (*p* = 0.007) in terms of diarrhoea and nausea and vomiting. There was also a significant difference in appetite loss between groups one and two (*p* = 0.000) and between groups one and three (*p* = 0.000).

**TABLE 5:** Cervical cancer specific symptoms reported by the respondents per group (*n* = 153).

Variables	Mean ranks			Overall mean	Standard deviation	*p*
	M0 (*n* = 51)	M6 (*n* = 51)	M12 (*n* = 51)			
Gastrointestinal symptoms						
Diarrhoea	37.3	24.2	6.5	22.7	31.7	0.000*
Appetite loss	43.1	7.2	9.8	20.0	29.5	0.000*
Constipation	22.2	6.5	14.4	14.4	28.3	0.076
Nausea and vomiting	21.9	6.2	2.6	10.2	24.8	0.000*
Urologic symptoms						
Urine frequency	52.3	32.0	50.3	44.9	34.3	0.004*
Burning sensation when urinating	40.5	24.8	26.1	30.5	33.8	0.054
Urine leakage	10.5	8.5	12.4	10.5	22.4	0.625
Difficulty emptying the bladder	9.8	5.9	5.9	7.2	19.1	0.552
Gynaecological symptoms						
Menopausal symptoms (hot flushes and/or night sweats)	29.4	20.3	22.9	24.2	27.6	0.151
Vaginal discharge	30.7	11.1	15.7	19.2	38.2	0.000*
Vaginal bleeding	19.0	2.0	6.5	9.2	23.6	0.000*

* , statistically significant, *p* ≤ 0.05.

When examining the urological data, difficulty in emptying the bladder had the lowest mean score (*m* = 7.2) and urinary frequency the highest (*m* = 44.9). In addition, urinary frequency had the highest mean score of all the symptoms investigated across the groups. There was no statistically significant difference in burning sensation when urinating, urine leakage and difficulty emptying the bladder; however, there was a statistically significant difference in urine frequency. A *post hoc* test showed there was a significant difference between groups one and two (*p* = 0.008) and between groups two and three (*p* = 0.010).

Vaginal bleeding was the gynaecological symptom with the lowest overall mean (*m* = 9.2), while menopausal symptoms, hot flushes and/or night sweats had the highest mean (*m* = 24.2). With regard to menopausal symptoms, there was no significant difference among the three groups; however, according to a *post hoc* analysis, there was a statistically significant difference in vaginal discharge and vaginal bleeding. Regarding vaginal discharge, there was a significant difference between groups one and two (*p* = 0.000) and groups one and three (*p* = 0.000). There was also a significant difference between groups one and two (*p* = 0.000) and between groups one and three (*p* = 0.000) in terms of vaginal bleeding.

Lastly, sexual functioning was investigated, specifically sexual activity, sexual enjoyment and vaginal functioning. There was statistically significant difference in sexual activity, sexual enjoyment and vaginal functioning among the three groups. A *post hoc* analysis indicated there was a significant difference in sexual activity between groups one and two (*p* = 0.000) and between groups one and three (*p* = 0.000). There were significant differences in sexual enjoyment between groups one and two (*p* = 0.000) and between groups one and three (*p* = 0.000). There were statistically significant differences in vaginal functioning between groups one and two (*p* = 0.000) and between groups one and three (*p* = 0.000) (Table 6).

**TABLE 6:** Sexual functioning reported by the different groups (*n* = 153).

Variable	Mean ranks			Overall mean	Standard deviation	Sig.*
	M0 (*n* = 2)†	M6 (*n* = 25)†	M12 (*n* = 30)†			
Sexual activity	96.7	71.9	62.1	76.9	35.5	0.000*
Sexual enjoyment	96.1	69.9	68.0	78.0	35.9	0.000*
Vaginal functioning	98.4	80.5	78.9	86.0	22.8	0.000*

* , statistically significant, *p* ≤ 0.05.

† , *n* = 2, *n* = 25, and *n* = 30 sample size varied because only those who were sexually active gave responses for sex-related questions.

## Discussion

The age profile of the sample seems to be typical of women treated for cervical cancer. Snyman and Herbst (2013) in a South African study investigating late presentation found that the mean age of their sample was 53 years compared to the 50.6 years in this study. Kaila and Maree (2018) in a study conducted at the same setting than this study found a similar trend with a mean age of 50.3 years. What differs is that the youngest respondent in this study was 30 years old, while the youngest respondent in the other two studies was 24 and 26 years, respectively.

The study provided evidence that slightly more than half of the women suffered from stage IIIA and more advanced cervical cancer. Although a similar trend was reported in a Polish study (Pasek, Suchocka & Urbański 2013), this percentage is about 30% less than what Snyman and Herbst (2013) found in their study, conducted at another academic hospital in Gauteng. The reason for this difference is unknown; however, it could be possible that the women in this study were primarily from urban areas and not rural areas as found in the previous study, as South African women from rural areas are less aware of cervical cancer and tend to consult traditional healers about abnormal vaginal bleeding (Pillay 2002). However, the drawbacks of living in a rural area are not limited to South Africa, as Palacio-Mejía and others (2003) in a study conducted in Brazil described the disadvantages of rural women compared to urban women in surviving cervical cancer.

It was positive to find that the global health status of the groups improved significantly from the time they received treatment to 12 months after treatment. Du Toit and Kidd (2015), in a South African study, found a similar trend when comparing the QOL of South African women who received radiotherapy alone to those who received chemoradiation. The global health status of both groups improved significantly from before treatment to 3 months after treatment. In addition, Ferrandina and colleagues (2012), in a longitudinal study conducted in Italy, found the global health status of women with locally advanced cervical cancer treated with radiotherapy or chemoradiation plus radical surgery improved gradually but significantly from before treatment to 12 months after surgery. What is interesting is that the baseline mean global health score of the Italian group was higher than the current group who was receiving treatment (72.9 vs. 57.5), while the current 12 months after treatment group had a slightly higher score compared to the Italian group 12 months after surgery (82.4 vs. 79.9). Smith, Avis & Assmann (1999) remind health care practitioners that patients consider physical functioning more important when appraising health status compared to QOL, where mental health is emphasised. It was interesting to find the physical functioning of the groups in this study did not improve significantly despite the significant improvement in their global health status.

None of the functioning scores showed a statistical significant difference between the groups. This is in contrast with the findings of Bjelic-Radisic et al. (2012) who, in a study investigating QOL in European women, found a significant difference in all the functioning scales between the groups on treatment and those who were followed up, similar to what Du Toit and Kidd (2015) found. What is noteworthy is that social functioning scored the lowest of the functional scores, while emotional functioning scored the lowest among European women (Bjelic-Radisic et al. 2012; Pasek et al. 2013). Studies conducted in the African context (Maree & Kaila 2014; Maree, Langley & Nqubezelo 2015; Van Schalkwyk, Maree & Wright 2008) found that women experience severe vaginal bleeding, sometimes offensive and difficult to manage, prior to being treated for cervical cancer, which leads to embarrassment and social isolation. In addition, these women are also stigmatised because of the bleeding, the smell, their diagnosis and fear of being infected with cervical cancer, which could explain the low social functioning scores. Social isolation has a devastating influence on QOL as African culture supports a philosophy of ‘Ubuntu’, which does not define a person in terms of individual characteristics, but in terms of their cultural traditions and social bonds (Mabovula 2011).

Financial difficulties, especially during the time of treatment, had a negative influence on QOL. This does not seem to be unique to Africa, as, although not to the same extent, studies conducted across Europe and in Turkey and Poland report financial difficulties in this group of women especially during the treatment phase (Bjelic-Radisic et al. 2012; Krikeli et al. 2011; Pasek et al. 2013; Yavas et al. 2017). Although treatment is free of cost to South African patients, women with cervical cancer have to travel to the hospital on a daily basis during the time of their treatment, which could explain this finding. In addition, persistent financial difficulties are not new to the South African context, as more than 40% of people in the same age group as the respondents in this study live under the upper bound poverty line (Stats SA 2017). Ntinga and Maree (2015) in a study focusing on the late effects of cervical cancer and its treatment found that women 12 months after treatment experience various health problems for which they are not treated at primary health clinics because of their cancer diagnosis. They are referred to the hospital where their cancer was treated, which could be one of the reasons why those in the 12 months after treatment group reported a higher financial burden compared to the 6 months after treatment group.

It was interesting to find that insomnia was the most troublesome general symptom, followed by pain and fatigue. Goker and others (2011) confirm this finding but report that fatigue was the most prevalent problem for Turkish women treated for cervical cancer followed by pain and insomnia. Pasek and colleagues (2013), in a Polish study comparing QOL in women during treatment, after treatment and 5–6 months after completing treatment also found insomnia had the highest mean score across the groups followed by fatigue and diarrhoea with pain in the fourth place. What was positive is that the three most prevalent symptoms in this study, insomnia, pain and fatigue, showed a downward trend from during treatment to 6 months after treatment, while these symptoms showed an upward trend in the Polish study. Despite this downward trend, Ntinga and Maree (2015) confirmed that pain and fatigue, as long-term complications of cervical cancer and its treatment, have a negative influence on QOL and hinder women from performing their normal activities of daily living.

What is of concern is the significant upward trend of swelling of the feet as lymphoedema is a well-known complication of pelvic radiotherapy. Heijkoop and others (2017) report an upward trend in lymphoedema from the time of receiving treatment until 12 months after treatment. However, it does not seem as if this change is significant. Khutjwe (2018) in a South African study investigating the prevalence of lymphoedema in women treated for gynaecological cancer found more than 30% of these women reported swelling of the lower limb, while 29.7% had lymphoedema. Lower limb lymphoedema is associated with a lower QOL, less satisfaction with functional well-being and insomnia (Shaitelman et al. 2015) which could have added to the fact that insomnia remained a distressing problem across the groups in this study.

Urinary frequency was the most troublesome cervical cancer-related symptom experienced accompanied by a burning sensation, most commonly occurring in the treatment phase. A study conducted in the Netherlands by Heijkoop and others (2017) supports these findings and, similar to this study, reports a ‘small improvement’ in urinary frequency in the period of 3–12 months after treatment. Lind and others (2011), in a study conducted among long-term cervical cancer survivors in Sweden, also found urinary symptoms had the highest mean score of all the symptoms investigated. In addition, qualitative studies (Dzaka & Maree 2016; Ntinga & Maree 2015) explain the negative effects these symptoms have on the lives of women not only during treatment but also in the long term.

The study provided evidence that all the domains of sexual functioning, sexual activity and enjoyment and vaginal functioning declined after treatment. However, when the short-term and late effects of cervical cancer and its treatment are considered, this finding does not seem surprising. It was interesting to note that other studies found a similar trend except for sexual activity, for instance Rahman and others (2017), in a study conducted in India, found the mean score for sexual activity increased from before treatment to 6 months after treatment with sexual enjoyment and vaginal functioning decreasing. Similarly, Heijkoop and others (2017) found sexual activity showed a ‘medium improvement’ from before treatment to 12 months after treatment, while sexual enjoyment showed a ‘small deterioration’. The reason why the sexual activity of this study population did not improve after treatment is not known; however, in addition to the physical changes, factors such as sexual activity before diagnosis and treatment, marital status and age could also play a role but this would need investigation before conclusions could be made.

### Limitations

The study has various limitations. Using a cross-sectional design did not allow for the investigation of changes in the same respondents over time. A longitudinal design may have resulted in results that were more accurate. In addition, respondents could have provided socially acceptable answers and not reflect what is true for them. However, the researchers believe the study provided base line data regarding the QOL of women treated for cervical cancer.

### Implications for nursing practice

Nurses practising in radiation oncology care settings should be cognisant of how cervical cancer and its treatment influence the QOL of women. Assessing the QOL during treatment and follow-up visits would allow nurses to develop interventions to address distressing problems timeously. In addition, nurses in Africa should assess social functioning before and after treatment and develop programmes to assist women to optimise their functioning as social beings despite having cervical cancer and prevent social dysfunction.

## Conclusion

Despite an improvement in the global health status, cervical cancer and its treatment had a negative influence on the QOL of the women and influenced all domains of their lives. Social functioning was the most affected and did not improve significantly over time. Financial difficulties were troublesome, especially during the time of treatment; urinary frequency and insomnia remained problematic; and swelling of the feet increased significantly over time.
